# Epidemiology of first cases of SARS-CoV-2 infection, from March to April 2020, in Gabon

**DOI:** 10.12688/f1000research.74378.2

**Published:** 2022-11-03

**Authors:** Sonia Etenna LEKANA-DOUKI, Nadine N'DILIMABAKA, Elvire MBONGO-KAMA, Marisca KANDET YATTARA, Armel MINTSA NDONG, Audrey Michel NGONGA DIKONGO, Julia Cyrielle ANDEKO, Ornella ZONG MINKO, Danielle Styvie KOUMBA MAVOUNGOU, Abdoulaye DIANE, Arsene MABIKA MABIKA, Telstar NDONG MEBALEY, Nal Kennedy NDJANGANGOYE, Octavie BANGA MVE-ELLA, Linda BOHOU KOMBILA, Joa Braithe MANGOMBI PAMBOU, Jeordy Dimitri ENGONE ONDO, Gael Darren MAGANGA, Jean-Bernard LEKANA-DOUKI

**Affiliations:** 1 Centre Interdisciplinaire de Recherches Medicales de Franceville, Franceville, BP769, Gabon; 2 Université de Masuku, Franceville, BP901, Gabon; 3 Hôpital d'Instruction des Armées Omar Bongo Ondimba, Libreville, Gabon; 4 Hôpital d'Instruction des Armées d'Akanda, Libreville, Gabon; 5 Laboratoire National de Santé Publique, Libreville, BP2215, Gabon; 6 Institut National Supérieur d'Agronomie et de Biotechnologie, Franceville, BP931, Gabon; 7 Université des Sciences de la Santé, Libreville, BP4009, Gabon

**Keywords:** COVID-19, Diagnostic, Epidemiology, Viral load, S gene, Gabon

## Abstract

Background

After the first cases of coronaviruses disease 2019 (COVID-19) in China in January 2020, the epidemic spread around the world. Few data are available from Central Africa. We conducted a study to monitor this emerging disease in Gabon, a Central Africa country.

Methods

In order to set up an epidemiological surveillance of COVID-19 in Gabon, we led molecular investigations on nasopharyngeal and oropharyngeal samples from the 1161 first suspected cases of COVID-19. A Reverse Ttranscriptase Polymerase Chain Reaction (RT-PCR) was performed using primers and probes targeted the E gene and polymerase gene according to the kit Tib-Molbiol.

Results

We diagnosed the first case of COVID-19 on March, 12 2020. Among those suspected cases, 83 were confirmed cases. There was no significant difference in prevalence of SARS-CoV-2 between age groups (p = 0.14). Seventy-three percent were asymptomatic. The viral loads were significantly higher in the nasopharyngeal samples than in the oropharyngeal samples (p=0.03). There was no significant difference in viral loads between age groups (p=0.9895) and no correlation between clinical symptoms and viral loads (p=0.06042).

Conclusion

In conclusion, this study provides the first molecular data from Gabon concerning the COVID-19 pandemic. The data showed that most of the infected people were asymptomatic. The viral load was higher in the nasopharyngeal samples.

## Introduction

In December 2019, an atypical pneumonia emerged in China. A total of 44 case-patients were reported from December 31, 2019, to January 4, 2020, with an unknown etiology ( https://apps.who.int/iris/handle/10665/330760). In January 2020, a 2019 novel coronavirus was identified, and named severe acute respiratory syndrome coronavirus 2 (SARS-CoV-2). SARS-CoV-2 caused an outbreak in China and rapidly spread in the whole world. ^1^ From January 20, 2020, the first cases were reported in North America. ^2^ In Europe on January 24, 2020, the first cases were reported by the World Health Organization (WHO). ^3^ On March 11, the WHO declared COVID-19 a pandemic disease ^4^ and reported 118,319 confirmed cases and 4292 deaths ( https://www.who.int/docs/default-source/coronaviruse/situation-reports/20200311-sitrep-51-covid-19.pdf). In Central Africa, Gabon reported its first case of COVID-19 on March 12, 2020. On December 15, 2020, nearly a year after its discovery, this disease continues to rise in the world with 70 million cumulative cases and 1.6 million deaths globally since the beginning of the pandemic ( https://www.who.int/publications/m/item/weekly-epidemiological-update---15-december-2020).

The literature reports that COVID-19 has an incubation period of 1–14 days and it affects both children and adults. Symptoms present in children are similar to those found in adults with an incubation period of 1–14 days; however, over 90% of affected children are asymptomatic or have no severe form of the disease. ^4^ The SARS-CoV-2 virus is detected in several clinical samples, such as nasopharyngeal samples, oropharyngeal samples, sputum, saliva, serum, urine and stool. ^5^^,^^6^ The viral load can vary between the different clinical samples, throughout the infection after the onset of the first symptoms or could depend on the severity of the disease. ^5^^,^^6^ The study of the infectivity of SARS-CoV-2 includes the analysis of viral loads, the host’s immune response but also the genomic analysis of the strains circulating in order to provide a response to the pandemic and develop a vaccine candidate. ^7^^–^^9^

15 first mutations have been identified in the genome, particularly in the main sequence, in the genes encoding non-structural protein, the polymerase, the spike glycoprotein (S), the membrane glycoprotein, and the nucleocapsid phosphoprotein. ^10^ The mutation D614G in the gene coding S protein could be associated with a more virulent variant leading to a higher mortality rate ( http://dx.doi.org/10.1038/s10038-020-0808-9). More recently, several mutations were identified in the spike region gene in a new variant of SARS-CoV-2 in the United Kingdom and South Africa ( https://www.who.int/csr/don/21-december-2020-sars-cov2-variant-united-kingdom/en/).

The first cases of COVID-19 have been widely documented in many countries. However few data are available from sub-Saharan Africa, and more particularly from Central Africa. The aim of this study is to report epidemiological and molecular data on the SARS-CoV-2 virus.

## Methods

### Study design, patients information’s and samples

When the worldwide coronavirus pandemic began in January 2020, the “ *Centre Interdisciplinaire de Recherches Médicales de Franceville*” (CIRMF) set up a surveillance of COVID-19 in Gabon, according to the guidelines of the Gabonese Ministry of Health, the WHO Region Office for Africa and the Africa Center for Disease Control (Africa CDC). Patients who were enrolled in this study visited health centers for respiratory syndrome comprising fever (≥38°C) and runny nose, or fever and cough, or fever and sore throat. Epidemiological data, including the name, age, sex, and travel history during the month before onset and clinical data were collected. Other people were asymptomatic but had been in contact with people who tested positive for COVID-19. So, from February 2020, nasopharyngeal and oropharyngeal samples were collected from suspected cases in hospitals: the “ *Hôpital d’instruction des Armées Omar Bongo Ondimba*” (HIAOBO), the “ *Hôpital d’instruction des Armées d’Akanda*” (HIA A) in Libreville, mobile health centers which collected samples, and health centers in the Haut-Ogooue province. From February 15 to April 30, 2020, 1161 first suspected cases were enrolled in Libreville, Franceville, Port-Gentil and Bitam for the confirmation of SARS-CoV-2 infection with molecular test diagnostics. The size of the study had correlated with samples collected for a month and a half after the first positive case. After collection, samples were stored in transport medium at 4°C until they were sent to the CIRMF along with the patient’s medical history mentioned demographical and clinical data such as symptoms and signs of the disease, comorbidities and travel history. The lack of information about age on some files constituted a bias.

### Molecular diagnosis and viral load of SARS-CoV-2

The confirmation of SARS-CoV-2 was done by real-time reverse transcriptase polymerase chain reaction (RT-PCR) according to Africa CDC guidelines. Swabs samples processed in a biosafety level-3 laboratory with personal protection equipment were placed in saline (0.9%). RNA extraction was done with the QIAamp ^®^ Viral RNA mini Kit (Qiagen) according to the manufacturer’s instructions. An extraction control was introduced during this step allowing the validation of the diagnosis by amplifying a gene fragment from Equine Arteritis Virus according to the instructions of the “TIB MOLBIOL” kit. An extraction control was introduced during this step. RT-PCR was performed using Superscript III RT-PCR kit Invitrogen in simplex, primers and probes targeted the E gene coding envelope and the RdRP gene coding RNA-dependent RNA polymerase (kit Tib-Molbiol), in a 7500 Real Time PCR System (Applied Biosystems). ^11^ The samples were considered negative if the cycle threshold (Ct value) exceeded 36 cycles for the E gene and 40 cycles for the RdRP gene. A person was confirmed as positive case if both nasopharyngeal and oropharyngeal sample were positive or if one of the two was positive for SARS-CoV-2. Viral load was calculated from the Ct value using the standard curve generated by dilution of RNA positive control.

### Statistical analysis

Patient records were used as the data sources. Data were analyzed using the Statview version 5.0 software. Pearson’s Chi-squared test and Fisher’s exact test were used to compare variables and to assess the relation between demographical data such as sex, age and clinical data. The R software package (version 4.0.3) was also used to compare the viral load between nasopharyngeal and oropharyngeal clinical samples, the viral load among asymptomatic patients and those with symptoms and the viral load across age groups. Data analysis revealed that the distribution did not follow a normal distribution. So a non-parametric analysis with with the Mann–Whitney U test. A p-value less than 0.05 was considered statistically significant.

## Results

### Epidemiological data of suspected and confirmed cases

This study took place between February 15, 2020, and April 30, 2020. The first cases of COVID-19 were detected on March 12, 2020, in Gabon. Among the 1161 suspected cases sampled, 517 (48.0%) were male, 560 (52.0%) were female. The sex ratio was 0.92, the median age was 36.0 years (range, 15 days to 82 years) and the mean age was 35.9±12.2 years ( Table 1). Information on gender and age was unavailable for 84 and 282 people, respectively, because some data were confidential at the beginning of the pandemic. The most represented age groups were 25–35 and 35–45 years, 32.4% and 34.4%, respectively, while the least represented group was composed of patients aged over 65 years (1.4%) ( Table 1). 96% of nasopharyngeal and oropharyngeal samples from the 1161 suspected cases were collected in the capital Libreville, and 19 (1.6%), 10 (0.9%), 16 (1.4%) were collected in Franceville, Port-Gentil and Bitam respectively ( Table 1).

**Table 1.** Demographic characteristics of suspected cases and prevalence of confirmed COVID-19 cases.

Characteristics	Suspected cases		Confirmed cases	
	n (%)	95% CI	N(p)	95% CI
**Sex**				
Male	517 (48.0)	45.0-51.0	40 (7.74)	5.38-9.98
Female	560 (52.0)	49.0-55.0	43 (7.68)	5.53-9.95
ND	84			
**Mean age**	35.9 ± 12.2	-	37.1 ± 12.9	-
**Median age**	36.0	-	37.5	-
**Interquartile**	13.0	-	12.0	-
**Age group (years)**				
[0-15]	59 (6.7)	5.0-8.4	7 (11.9)	3.6-20.2
[15-25]	47 (5.3)	3.8-6.8	0 (0.0)	-
[25-35]	285 (32.4)	29.3-35.5	20 (7.0)	4.0-10.0
[35-45]	302 (34.4)	31.3-37.5	27 (8.9)	5.7-12.1
[45-55]	136 (15.5)	13.1-17.9	16 (11.8)	6.4-17.2
[55-65]	38 (4.3)	2.9-5.7	2 (5.3)	-1.8-12.4
[65-82]	12 (1.4)	0.6-2.2	2 (16.7)	-4.4-37.8
ND	282	-	9	-
**Towns (Province)**				
Libreville (Estuaire)	1116 (96.1)	95.0-97.2	82 (7.4)	5.9-8.9
Franceville (Haut-Ogooué)	19 (1.6)	0.9-2.3	0 (0.0)	-
Port-Gentil (Ogooué-Maritime)	10 (0.9)	0.4-1.4	0 (0.0)	-
Bitam (Woleu- Ntem)	16 (1.4)	0.7-2.1	1 (6.2)	-5.6-18.0
**TOTAL**	**1161**	-	**83 (7.1)**	**5.6-8.6**

CI: confidence interval, ND: no data, (p): prevalence.

Among the 1161 suspected cases, 83 (7.15%) were confirmed cases of COVID-19. There was no significant difference between males (48.2%) and females (51.8%) according to prevalence, 7.74 and 7.68, respectively ( *X* ^2^=0.001, p=0.97). The median age was 37.5 years (range, 3 years to 68 years) and the mean age was 37.1±12.9 years ( Table 1). Regarding to the age group distribution, the highest percentages were in the 25–35 (24.1%) and 35–45 years (32.5%) age groups. However, there was no significant difference in prevalence of SARS-CoV-2 between age groups ( *X* ^2^=9.58, p=0.14). No confirmed cases were found in the 47 suspected cases of the 15-25 years age group ( Table 1). All the positive samples were collected in Libreville, except, one which came from Bitam.

### Clinical characteristics of patients with COVID-19

73% of the confirmed cases were asymptomatic ( Table 2). However the prevalence of COVID-19 in patients with a respiratory syndrome (18.9%) was higher than in asymptomatic cases (5.8%), ( *X* ^2^=25.17, p<0.0001). Among the 22 symptomatic cases, 20 (90.9%) had an influenza-like illness (ILI) defined as fever (≥38°C) and a runny nose, cough, or sore throat while two (9.1%) patients had respiratory distress. ILI symptoms were accompanied by anosmia and ageusia for two patients, one patient suffered from diarrhea and another had asthenia ( Table 2). 94% of people infected with the coronavirus did not have comorbidity. One confirmed case had asthma, two had sinusitis, one had diabetes and another had hypertension ( Table 2). The positive rate in people with chronic disease and no chronic disease was 6.3% and 7.2%, respectively. So, no association was found between comorbidities and SARS-CoV-2 infection ( *X* ^2^=0.004, p=0.95).

**Table 2.** Clinical characteristics of confirmed COVID-19 cases.

	n (%)	95% CI
**Clinical characteristic**		
Asymptomatic	61 (73.5)	64.0-83.0
Symptomatic	22 (26.5)	17.0-36.0
**Symptoms**		
Influenza-like-illness	16 (19.3)	10.8-27.8
Influenza-like-illness + anosmia + ageusia	2 (2.4)	-0.9-5.7
Influenza-like-illness + diarrhoea	1 (1.2)	-1.1-3.5
Influenza-like-illness + asthenia	1 (1.2)	-1.1-3.5
Respiratory distress	2 (2.4)	-0.9-5.7
**Chronic comorbidities**		
Asthma	1 (1.2)	-1.1-3.5
Sinusitis	2 (2.4)	-0.9-5.7
Diabetes	1 (1.2)	-1.1-3.5
Hypertension	1 (1.2)	-1.1-3.5
None	78 (94.0)	88.9-99.1
**TOTAL**	**83**	-

CI: confidence interval.

### Recent history of travel and context of COVID-19 screening

**Figure 1.**
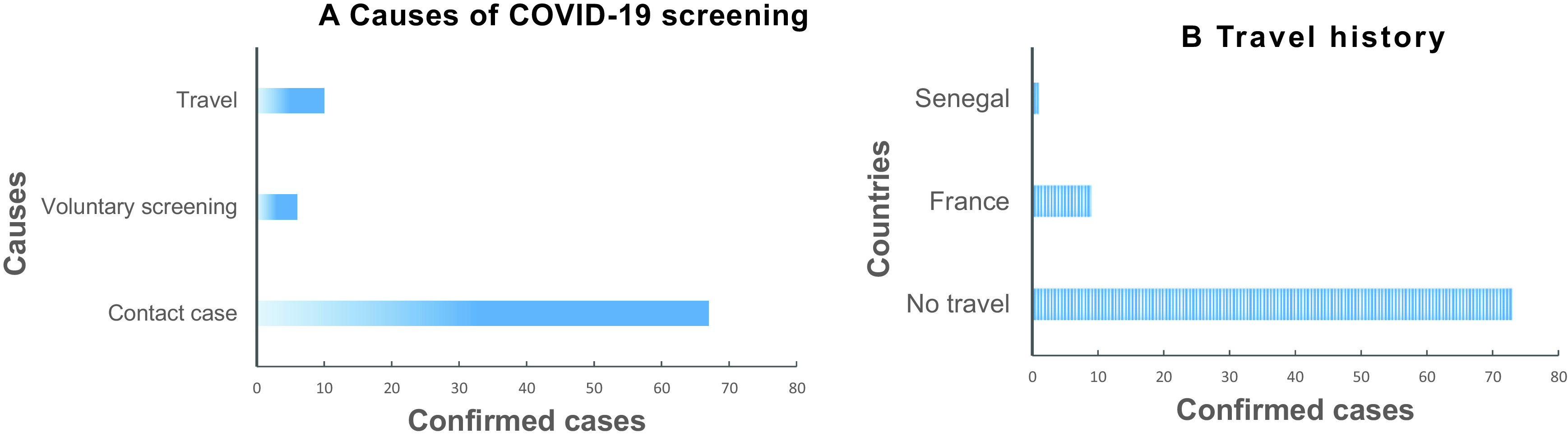
(A-B): Context of COVID-19 screening. A. Causes of COVID-19 screening. B. Travel history of confirmed cases.

The surveillance of COVID-19 in Gabon revealed that more than three-quarters of positive cases (87.9%) had no recent travel history, 67 (80.7%) were in contact with confirmed cases, and 6 (7.2%) persons participated in voluntary screening ( Figure 1A). The 10 (12.1%) people who had traveled from affected areas came from France (n=9) and Senegal (n=1) ( Figure 1B).

The prevalence was similar in the three categories of confirmed cases, the contact cases (6.7%), the voluntary screening cases (6.3%) and people who reported having traveled recently (13.7%), ( *X* ^2^=5.07, p=0.08). All positive cases who presented themselves for voluntary screening were asymptomatic, whereas all the confirmed cases who had a recent travel history abroad had respiratory symptoms. Among the 67 contacts cases 55 were asymptomatic.

### Viral load of SARS-CoV-2 in oropharyngeal and nasopharyngeal clinical samples

**Figure 2.**
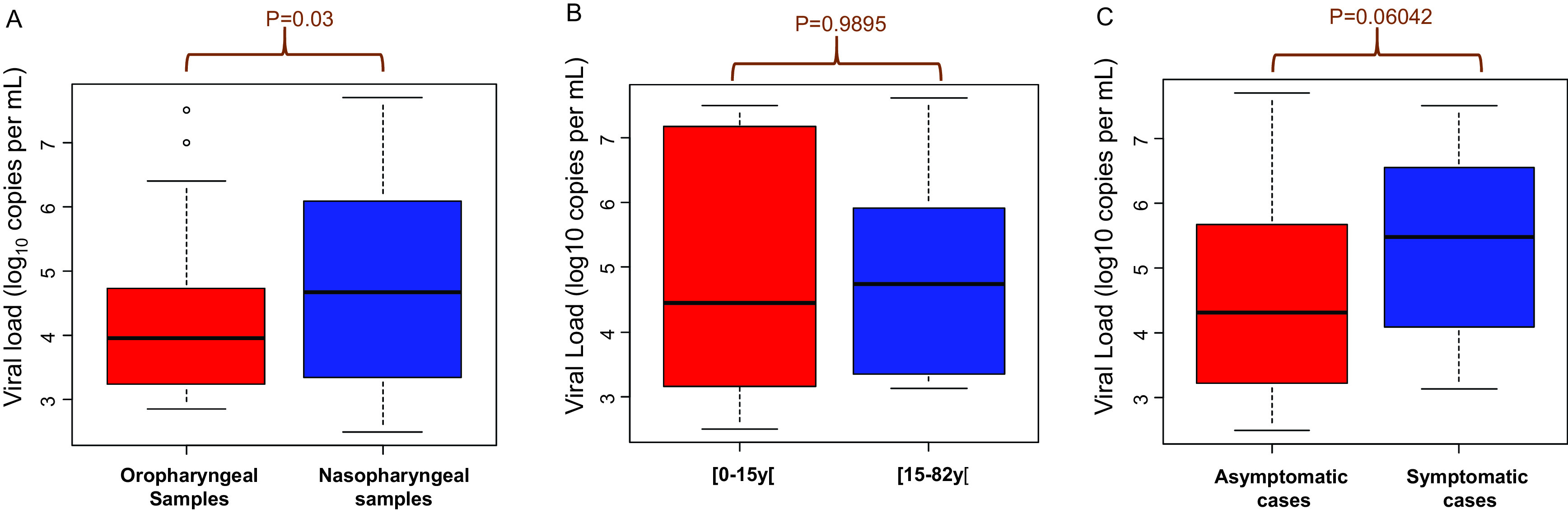
(A-C): Viral load of SARS-CoV-2 in clinical samples. A: Viral load from oropharyngeal and nasopharyngeal samples. B: Comparison of viral loads in two age groups. C: Comparison of viral loads in symptomatic and asymptomatic people.

The viral loads in oropharyngeal samples ranged from 2.86 log _10_ copies/mL (7.25×10 ^3^ copies/mL) to 7.51 log _10_ copies/mL (3.21×10 ^7^ copies/mL) with a median of 3.96 log _10_ copies/mL (9.13×10 ^3^ copies/mL) ( Figure 2A). In the nasopharyngeal samples the viral load ranged from 2.50 log _10_ copies/mL (3.16×10 ^3^ copies/mL) to 7.70 log _10_ copies/mL (4.98×10 ^7^ copies/mL) with a median of 4.68 log _10_ copies/mL (47.67×10 ^3^ copies/mL). The viral loads were significantly higher in the nasopharyngeal samples than in the oropharyngeal samples (p=0.03) ( Figure 2A).

There was no significant difference in viral loads between children under 15 years old and people over 15 years old (p=0.9895) ( Figure 2B). Likewise, there was no correlation between clinical symptoms and viral loads (p=0.06042) ( Figure 2C).

## Discussion

Between, March 12, 2020, and April 30, 2020, 83 confirmed cases of COVID-19 were detected in Gabon. During the 50-day period, the progression of the disease was slow and comparable to the spread of COVID-19 in Nigeria during the first 45 days. However, in several countries in Africa, such as Cameroon, South Africa, Tunisia, Morocco, the burden of COVID-19 was higher, with more than 500 confirmed cases reported 35 days after the first case. ^12^ This difference could probably due to the population density which would be higher in these countries. Several studies which described more confirmed cases showed that more than three quarters of patients were found in the [30–79] age group. ^13^^,^^14^ Despite the fact that all ages were susceptible, others studies showed that the majority of cases were over 30 and more specifically between 30 and 60 years old. ^14^^,^^15^ The 30-years-olds in our study were one of the most represented age groups (32.4%).

More than 70% of the cases were asymptomatic in our study. Two studies which reported the first cases of COVID-19 in China and Europe mentioned that 5% of case were asymptomatic. ^3^^,^^16^ The proportion of asymptomatic cases varied according to the studies, values ranged from 1% to 78% ^7^^,^^17^^,^^18^ ( http://dx.doi.org/doi:10.1136/bmj.m1375). The percentage of asymptomatic cases (73.5%) in Gabon during this period was within the range of the value reported worldwide meaning that asymptomatic carrier transmission was effective in Gabon. Moreover, there may have been presymptomatic transmission in Gabon such as the one described in Gernamy where an asymptomatic businessman from China transmitted the virus to a healthy German businessman. The Chinese visitor reportedly had symptoms of COVID-19 upon his return. ^19^ Several reports cases have recorded similar facts and shown asymptomatic and presymptomatic transmission. ^20^^,^^21^ Approximately 98% of patients would had an incubation period of 5 days on average ranging from 2 to 14 days, although this period could be up to 24 days. ^4^ These data could explain the spread of the virus in Gabon despite the high number of asymptomatic cases (73%).

Review authors described symptoms of COVID-19 as predominantly flu-like symptoms such as fever (80–90%), cough (50%), lethargy (20–40%) and in some cases diarrhea. ^4^ In some more vulnerable patients including the elderly and people with chronic diseases, the symptoms progress to pneumonia and respiratory distress, requiring in 20% of cases hospital treatment. ^4^ In addition, 80% of COVID-19 infections in China were mild flu-like symptoms. ^4^ Our data corroborated those of this study. Indeed, a large number of symptomatic patients developed mild flu-like symptoms (90% of symptomatic patients) ( Table 2). Only two patients had respiratory distress and 78 (94%) had no chronic disease ( Table 2). However, since our data only considered the first cases in Gabon, the number of cases was low and probably underestimated.

In the 50 days following the report of the first case of COVID-19 on March 12, 2020, 87% of confirmed cases declared that they had not made a recent trip ( Figure 1A), which would suggest that the virus was introduced in Gabon before March. The WHO declared the COVID-19 pandemic on March 11, 2020 meaning that a large number of countries across the African continent were affected by this period. The flow of travelers and international trade between Gabon and various affected countries would explain the introduction of the virus in Gabon. The slow spread is said to be linked to the fact that the government acted by closing borders, closing schools, quarantine of all suspected and confirmed cases and restricting movement as early as the end of March.

Some studies report a very high SARS-CoV-2 viral load in nasal swabs and others in throat swabs. ^8^ We found that the viral loads were significantly higher in the nasopharyngeal samples than in the oropharyngeal samples (p=0.03) ( Figure 2A). Our data is similar to a study which compared the viral load in different clinical samples (saliva, spectrum, urine, nasopharyngeal and oropharyngeal samples) and showed that the viral load was the highest in the nasopharynx. ^5^ One review based on seven studies measured and compared the viral load in pre-symptomatic, asymptomatic and symptomatic patients. It reported little to no difference between the SARS-CoV-2 viral load in the three categories of patients. ^8^ Our results which showed no correlation between the viral load and the clinical symptom of patients are in accordance with these studies. Some studies compared the SARS-CoV-2 viral loads in patients of different age groups in order to establish a possible link between the viral load, the duration of symptoms and the age of the patients. They concluded that there was no significant difference with regard to viral load or the duration of virus detection between adults and children. ^8^ The number of patients in each age group and the relationship between viral load and infectivity should be considered. Our study shows that there is no difference between patients under 15 and patients over 15 ( Figure 2).

It is important to characterize the virus SAR-CoV-2 by identifying the genotype circulating in Gabon in order to set up an efficient response to this pandemic. The first SARS-CoV-2 genome of the isolate Wuhan-Hu-1 was sequenced in China (accession number NC_045512 or MN908947) in January 2020. The literature reported a frequent mutation in the S gene (position 23403A>G) coding the variant of S protein D614G ^10^ ( http://dx.doi.org/10.1038/s10038-020-0808-9). This mutation emerged in Europe and North America on January 29 and February 28, 2020 respectively. ^22^ Initially, the D614 and G614 variants reached an equal ratio in Europe and North America at the end of February, then in March, the G614 variant started to circulate predominantly in both continents from March. ^22^ We performed the whole genome of three of the samples from the first cases as part of a study on the genotyping of strains of SARS-CoV-2 circulating in Gabon. ^23^ These samples 34 (Accesion number GenBank MW512911, GISAID EPI_ISL_8886131), 56 (Accesion number GenBank MW512912, GISAID EPI_ISL_8886132) and 62 (Accesion number GenBank MW512913, GISAID EPI_ISL_8886133) belonged to lineage B.1, A, B.1.356 respectively. The introduction of lineage B was characteristic of the D614G mutation. These information corroborated that of a study which reported the emergence of the G614 variant in Africa on March 13, 2020. ^22^ At this time, the circulation of both the D614 and G614 variants in Europe and the world suggested a simultaneous introduction of the two strains in Gabon. A Japanese study showed a significant positive correlation between the S protein 614G variant and fatality rates ( http://dx.doi.org/10.1038/s10038-020-0808-9). They analyzed the frequency of mutations in the SARS-CoV-2 genome in 28 countries which they grouped into three clusters in which they show a correlation between the mutations and the fatality rate ( http://dx.doi.org/10.1038/s10038-020-0808-9). The whole genome of 64 Gabonese strains of the beginning of the pandemic provided information on the predominant lineage B.1.1 (51/64) of this period. ^23^

In conclusion, this study reported the first data on the COVID-19 pandemic in Gabon. The data showed that most of the infected people were asymptomatic. The viral load was higher in the nasopharyngeal samples. Biochemical and immunological investigations would provide additional data and increase knowledge about the circulation and the impact of the virus in Gabon and more generally in Africa.

## Data availability

BioStudies: “Epidemiology of first cases of SARS-CoV-2 infection, from March to April 2020 and molecular characterization of the spike protein, in Gabon”. Accession number S-BSST800: https://identifiers.org/biostudies:S-BSST800.

## Consent

Informed consent for publication of the participants/patients’ details was obtained from the participants/patients/parents/guardian/relative of the participant/patient.’

## Ethics declaration

This study was approved by the national ethics committee (N°0003/2020/CNER/SG/P) and was performed in accordance with the ethical standards of the Declaration of Helsinki of 1964. Consent was obtained before sampling.
