# Supplementary material for: Epidemiology of first cases of SARS-CoV-2 infection, from March to April 2020, in Gabon
Source: F1000Res. 2022 Nov 3;11:205. Originally published 2022 Feb 17. [Version 2] doi: 10.12688/f1000research.74378.2 (PMC9672532; doi:10.12688/f1000research.74378.2)
Supplement: Peer review history

# Peer review: Reviewer response for version 2

**DOI:** 10.5256/f1000research.140568.r154934

**Published:** 2022-11-17

**Authors:** Babatunde Motayo

**Affiliations:** 1 Department of Virology, College of Medicine, Federal Medical Centre Abeokuta, University of Ibadan, Ibadan, Nigeria

The authors have satisfied all my concerns; the paper can now be approved.

Is the work clearly and accurately presented and does it cite the current literature?

Partly

If applicable, is the statistical analysis and its interpretation appropriate?

Yes

Are all the source data underlying the results available to ensure full reproducibility?

Yes

Is the study design appropriate and is the work technically sound?

Yes

Are the conclusions drawn adequately supported by the results?

Partly

Are sufficient details of methods and analysis provided to allow replication by others?

Partly

Reviewer Expertise:

Virology; Molecular Epidemiology; Evolutionary Biology

I confirm that I have read this submission and believe that I have an appropriate level of expertise to confirm that it is of an acceptable scientific standard.

# Peer review: Reviewer response for version 1

**DOI:** 10.5256/f1000research.78125.r140050

**Published:** 2022-08-08

**Authors:** Anggraini Dwi Sensusiati

**Affiliations:** 1 Department of Radiology, Universitas Airlangga Hospital, Surabaya, Indonesia

This article is about the epidemiological data of the first SARS-Cov-2 infection case in Gabon and the molecular characteristics data of the spike protein. Based on the results of the study, it was explained that the first COVID-19 case in Gabon was found on March 12, 2020. The characteristics of confirmed COVID-19 patients are mostly asymptomatic. While symptomatic patients mostly have influenza-like symptoms (ILI symptoms), namely fever; runny nose; cough; sore throat; and some of them experience respiratory distress. Some patients with ILI symptoms are also accompanied by anosmia, ageusia, diarrhea, or asthenia. The results of the study also stated that there was no relationship between comorbidities and SARS-Cov-2 infection. Based on travel history, all samples confirmed to have COVID-19 who traveled abroad had respiratory symptoms.

 In the results of the study related to the molecular characteristics of spike proteins, it was explained that the viral load was higher in nasopharyngeal samples than in oropharynx; there was no significant difference in the viral load of children under 15 years of age with people aged 15 years and over; and there is no correlation between the clinical symptoms of confirmed COVID-19 patients and viral loads.

 Phylogenetic analysis in this study was carried out with five sequences of 3822 spike S gene nucleotides, obtained from 5 patients (2 symptomatic patients originally from Paris; the first case in Gabon; and 2 contact cases without travel history). The S genes that have been analyzed suggest the introduction of the D614 and G614 variants in Gabon.

 In general, this research is good. The results of the study are important to enrich data and literature related to the epidemiology of COVID-19 in all countries around the world, including Gabon. The results of molecular research of the virus are also useful to enrich the genome database of the COVID-19 virus, which can be used for other molecular research in the future.

 The systematic of writing this article has also been good. The writing of research methods is good and complete. Especially in the study design section and patient information. The number of samples used is also sufficient. The writing of the results and discussion have also answered the purpose of the study.

 To make the discussion section more comprehensive, it would be better if the authors add more information related to the profession and the activities of the sample. It is also necessary to add more detailed information related to comorbidities such as the level of blood sugar in diabetic patients.

Is the work clearly and accurately presented and does it cite the current literature?

Yes

If applicable, is the statistical analysis and its interpretation appropriate?

I cannot comment. A qualified statistician is required.

Are all the source data underlying the results available to ensure full reproducibility?

Yes

Is the study design appropriate and is the work technically sound?

Yes

Are the conclusions drawn adequately supported by the results?

Yes

Are sufficient details of methods and analysis provided to allow replication by others?

Yes

Reviewer Expertise:

COVID-19, Radiology, Laboratory Findings, Neuroradiology, and Head & Neck Imaging, Artificial Intelligence for COVID-19.

I confirm that I have read this submission and believe that I have an appropriate level of expertise to confirm that it is of an acceptable scientific standard.

# Response

**Published:** 2022-10-31

**Authors:** Sonia LEKANA-DOUKI

**Affiliations:** Centre Interdisciplinaire de Recherches Medicales de Franceville, Gabon

**Responses to reviewer 2**

**Manuscript title:** **Epidemiology of first cases of SARS-CoV-2 infection, from March to April 2020 and molecular characterization of the spike protein, in Gabon**

 We were unable to obtain some detailed data such as sugar levels in diabetic patients because some data were missed during the implementation of monitoring at the begining of the pandemic.

 We changed the title and removed the sequencing section to better accommodate the other reviewer's suggestions.

 Title: “Epidemiology of first cases of SARS-CoV-2 infection, from March to April 2020” (line 26).

 The keywords have been modified: “COVID-19, Diagnostic, Epidemiology, Viral load, Gabon” (line 52).

 Since the first data presented in this study, we have carried out another study on the complete genomes of samples from the first wave of the pandemic in Gabon (line 273, 286). Thus, we have deleted the paragraph on Sanger's method in the methods section as well as the paragraph SARS-CoV-2 sequences in the results. We have modified the discussion in this way Three samples for which we had done sequencing by the Sanger method were selected in this other study to obtain the complete genome. These were samples 34, 56 and 62. We have therefore added information on their lineage to the discussion (line 273). We deleted the Figure 3.

 As a result, the title of the study has been modified because the data concerning the characterization of the strains by the genotyping of the S gene are no longer provided in this study but constitute a comment in the discussion which provided additional information on the lineage of the first cases of COVID-19 in Gabon.

# Peer review: Reviewer response for version 1

**DOI:** 10.5256/f1000research.78125.r141590

**Published:** 2022-07-18

**Authors:** Babatunde Motayo

**Affiliations:** 1 Department of Virology, College of Medicine, Federal Medical Centre Abeokuta, University of Ibadan, Ibadan, Nigeria

I thank the handling Editor of the F1000research for selecting me as a reviewer for the paper titled “Epidemiology of the first cases of SARSCoV-2 infection from March to April 2020 and molecular characterization of the Spike protein in Gabon”. I have gone through the paper and have made a decision of major revision. My comments and suggested revision are outlined below.

 MAJOR COMMENTS

 ABSTRACT: The background section of the Abstract needs to be re-written to summarize actual events that led to the study. The method section is not explicit enough nothing was mentioned about how the study was carried out or actual techniques used such as RT-PCR and sequencing, please re-cast to reflect the actual techniques used to carry out the study.

 METHODS; The authors did not mention anything about ethical approval and consent, this should be stated clearly if it was sought. The authors did not apply the generally acceptable classification system to properly genotype the Gabonese isolates identified, that is the PANGO-lineage and Nexclade classification. These two genotyping tools should be done and reported appropriately. Also, the associated genomic data that was generated to generate sequence alignments and the phylogenetic tree is not enough to conclusively derive any significant phylogenetic relationship, considering the enormity of data already available in databases such as GenBank and GISAID, at least a reasonable number of sequences from each sub-region of Africa and major continents, including China should be included. The authors should update with more sequences from diverse SARSCoV Lineages including major variants of concerns, variants of interest, and relevant global sequences and re-do the alignment and ML tree construction. The authors are also advised to improve on the tree visualization the labels should be bolder and more informative, I suggest the authors make use of tree visualization tools such as FigTree, or ggTree for better representation, authors are also advised to construct an MCC time tree showing lineage assignments of the Gabonese isolates along with relevant circulating lineages in Africa and their dates of introduction.

 RESULTS: The results in the first two sub-sections should be merged since they both report the same thing in different sub-populations. The sub-heading of the last sub-section SARSCoV-2 sequences should be re-casted e.g Molecular characterization of S-gene of SARSCoV-2. The molecular data from the suggested analysis such as PANGO-lineage should be included also; results of phylogenetic analysis should reflect lineage clustering, geographic diversification from previous/more recent related isolates, e.t.c. A time tree will also show introductions of same lineage, or multiple lineages within the time frame under study.

 DISCUSSION: The discussion aspect has to be re-written to include the data generated from the suggested additional analysis particularly the genotype and potential implications. Also, the sample size of the sequences is too small and should be included as a limitation to the study. Also, other limitations should be clearly stated such as the inability to sequence the complete genome of the Gabonese SARSCoV-2 strains.

Is the work clearly and accurately presented and does it cite the current literature?

Partly

If applicable, is the statistical analysis and its interpretation appropriate?

Yes

Are all the source data underlying the results available to ensure full reproducibility?

Yes

Is the study design appropriate and is the work technically sound?

Yes

Are the conclusions drawn adequately supported by the results?

Partly

Are sufficient details of methods and analysis provided to allow replication by others?

Partly

Reviewer Expertise:

Virology; Molecular Epidemiology; Evolutionary Biology

I confirm that I have read this submission and believe that I have an appropriate level of expertise to confirm that it is of an acceptable scientific standard, however I have significant reservations, as outlined above.

# Response

**Published:** 2022-10-31

**Authors:** Sonia LEKANA-DOUKI

**Affiliations:** Centre Interdisciplinaire de Recherches Medicales de Franceville, Gabon

**Responses to reviewer 1**

**Manuscript title:** **Epidemiology of first cases of SARS-CoV-2 infection, from March to April 2020 and molecular characterization of the spike protein, in Gabon**

 Manuscript ID :

 Sonia Etenna Lekana-Douki ^a^, Nadine N’dilimabaka ^a,b^, Elvire Mbongo-Kama ^c^, Marisca Kandet Yattara ^d^, Armel Mintsa Ndong ^e^, Audrey Michel Ngonga Dikongo ^a^, Julia Cyrielle Andeko ^a^, Ornella Zong Minko ^a^, Danielle Styvie Koumba Mavoungou ^a^, Abdoulaye Diané ^f^, Arsène Mabika Mabika ^g^, Telstar Ndong Mebaley ^a^, Nal Kennedy Ndjangangoye ^a,h^, Octavie Banga Mve-Ella ^a^, Linda Bohou Kombila ^a^, Joa Braithe Mangombi ^a^, Jeordy Dimitri Engone Ondo ^f^, Gaël Darren Maganga ^a,i^, Jean-Bernard Lekana-Douki ^h,j^

***Reviewer 1***

***APPROVED WITH RESERVATIONS***

***I thank the handling Editor of the F1000research for selecting me as a reviewer for the paper titled “Epidemiology of the first cases of SARSCoV-2 infection from March to April 2020 and molecular characterization of the Spike protein in Gabon”. I have gone through the paper and have made a decision of major revision. My comments and suggested revision are outlined below*** ***.***

***MAJOR COMMENTS***

***ABSTRACT: The background section of the Abstract needs to be re-written to summarize actual events that led to the study. The method section is not explicit enough nothing was mentioned about how the study was carried out or actual techniques used such as RT-PCR and sequencing, please re-cast to reflect the actual techniques used to carry out the study.***

 We have re-written the section “ABSTRACT” with information about events that led to the study and information about le PCR and the sequencing (line 31 to 40).

***METHODS; The authors did not mention anything about ethical approval and consent, this should be stated clearly if it was sought.***

 We added the following information about ethical approved and consent in the section Ethics declaration **:**

 “This study was approved by the national ethics committee (N°0003/2020/CNER/SG/P) and was performed in accordance with the ethical standards of the Declaration of Helsinki of 1964. Consent was obtained before sampling.” (line 305).

***The authors did not apply the generally acceptable classification system to properly genotype the Gabonese isolates identified, that is the PANGO-lineage and Nexclade classification. These two genotyping tools should be done and reported appropriately. Also, the associated genomic data that was generated to generate sequence alignments and the phylogenetic tree is not enough to conclusively derive any significant phylogenetic relationship, considering the enormity of data already available in databases such as GenBank and GISAID, at least a reasonable number of sequences from each sub-region of Africa and major continents, including China should be included. The authors should update with more sequences from diverse SARSCoV Lineages including major variants of concerns, variants of interest, and relevant global sequences and re-do the alignment and ML tree construction. The authors are also advised to improve on the tree visualization the labels should be bolder and more informative, I suggest the authors make use of tree visualization tools such as FigTree, or ggTree for better representation, authors are also advised to construct an MCC time tree showing lineage assignments of the Gabonese isolates along with relevant circulating lineages in Africa and their dates of introduction*** ***.***

 Since the first data presented in this study, we have carried out another study on the complete genomes of samples from the first wave of the pandemic in Gabon (line 273, 286). Thus, we have deleted the paragraph on Sanger's method in the methods section as well as the paragraph SARS-CoV-2 sequences in the results. We have modified the discussion in this way. Three samples for which we had done sequencing by the Sanger method were selected in this other study to obtain the complete genome. These were samples 34, 56 and 62. We have therefore added information on their lineage to the discussion (line 273). We deleted the Figure 3.

 As a result, the title of the study has been modified because the data concerning the characterization of the strains by the genotyping of the S gene are no longer provided in this study but constitute a comment in the discussion which provided additional information on the first cases of COVID-19 in Gabon :

 “Epidemiology of first cases of SARS-CoV-2 infection, from March to April 2020” (line 26).

 The keywords have been modified: “COVID-19, Diagnostic, Epidemiology, Viral load, Gabon” (line 52).

***RESULTS: The results in the first two sub-sections should be merged since they both report the same thing in different sub-populations. The sub-heading of the last sub-section SARSCoV-2 sequences should be re-casted e.g Molecular characterization of S-gene of SARSCoV-2. The molecular data from the suggested analysis such as PANGO-lineage should be included also; results of phylogenetic analysis should reflect lineage clustering, geographic diversification from previous/more recent related isolates, e.t.c. A time tree will also show introductions of same lineage, or multiple lineages within the time frame under study*** ***.***

 We merged the first two sub-sections and changed the title of the sub-section: Epidemiological data of suspected and confirmed cases (line 146).

 We deleted the sub-section SARS-CoV-2 sequences for the reasons previously mentioned.

***DISCUSSION: The discussion aspect has to be re-written to include the data generated from the suggested additional analysis particularly the genotype and potential implications. Also, the sample size of the sequences is too small and should be included as a limitation to the study. Also, other limitations should be clearly stated such as the inability to sequence the complete genome of the Gabonese SARSCoV-2 strains*** ***.***

 We have re-written the discussion by mentioning the whole genomes made in another study which provides information on the lineages that circulated at the beginning of the pandemic (line 273, 286).
